# Histopathological Correlations between Mediastinal Fat-Associated Lymphoid Clusters and the Development of Lung Inflammation and Fibrosis following Bleomycin Administration in Mice

**DOI:** 10.3389/fimmu.2018.00271

**Published:** 2018-02-15

**Authors:** Yaser Hosny Ali Elewa, Osamu Ichii, Kensuke Takada, Teppei Nakamura, Md. Abdul Masum, Yasuhiro Kon

**Affiliations:** ^1^Department of Histology and Cytology, Faculty of Veterinary Medicine, Zagazig University, Zagazig, Egypt; ^2^Faculty of Veterinary Medicine, Basic Veterinary Sciences, Hokkaido University, Sapporo, Japan; ^3^Laboratory of Molecular Medicine, Faculty of Veterinary Medicine, Hokkaido University, Sapporo, Japan; ^4^Section of Biological Science, Chitose Laboratory, Japan Food Research Laboratories, Chitose, Japan; ^5^Department of Anatomy, Histology and Physiology, Sher-e-Bangla Agricultural University, Dhaka, Bangladesh

**Keywords:** bleomycin, lung inflammation, mediastinal fat-associated lymphoid cluster, mediastinal adipose tissue, C57BL/6 mice

## Abstract

Bleomycin (BLM) has been reported to induce lung inflammation and fibrosis in human and mice and showed genetic susceptibility. Interestingly, the C57BL/6 (B6) mice had prominent mediastinal fat-associated lymphoid cluster (MFALCs) under healthy condition, and showed susceptibility to development of lung fibrosis following BLM administration. However, the pathogenesis of lung lesion progression, and their correlation with MFALC morphologies, remain to be clarified. To investigate the correlations between MFALC structures and lung injuries in B6 mice, histopathological examination of mediastinal fat tissues and lungs was examined at 7 and 21 days (d) following a single 50 μL intranasal (i.n.) instillation of either BLM sulfate (5 mg/kg) (BLM group) or phosphate-buffered saline (control group). The lung fibrosis was examined by Masson’s trichrome (MT) stain of paraffin sections and mRNA expression levels of Col1a1, Col3a1, and Acta2 in different frozen lung samples. Furthermore, immunohistochemistry for CD3, B220, Iba1, Gr1, BrdU, LYVE-1, and peripheral node addressin (PNAd) was performed to detect T- and B-cells, macrophages, granulocytes, proliferating cells, lymph vessels (LVs), and high endothelial venules (HEVs). We found that MFALCs were more abundant in the BLM group as compared to the control group. The lung of BLM group developed pneumonitis with severe cellular infiltrations at 7 days and significant collagen deposition (MT) and higher expression of Col1a1, and Col3a1 at 21 days post-administration. Numerous immune cells, proliferating cells, HEVs, and LVs were observed in both MFALCs and lungs of the BLM group. Interestingly, PNAd + HEVs were observed in the lungs of the BLM group, but not the control group. Moreover, numerous Gr1 + polymorphonuclear and mononuclear-like ring cells were found in the MFALCs and lungs of the BLM group. Interestingly, flow cytometric analysis revealed a significant increase of B-cell populations within the MFALCs of BLM group suggesting a potential proliferative induction of B-cells following inflammation. Furthermore, significant positive correlations were observed between quantitative parameters of these immune cells in both the lungs and MFALCs. Thus, we suggest a potentially important role for MFALCs and HEVs in the progression of lung disease, especially in inflammatory lung disease.

## Introduction

Pulmonary fibrosis is one of the most life-threatening diseases due to its damaging effect on lung tissues, which then leads to organ failure. It is associated with considerable morbidity and mortality in humans (1, 2). The etiology of pulmonary fibrosis varies, whereas some chemicals such as bleomycin (BLM), fibrogenic environmental toxins, exposure to radiation, or other unknown factors could lead to its development (3). BLM is an antibiotic agent with antitumor activity that exerts its effect by inducing tumor cell death. Despite the development in the drugs thereby of oncology, BLM remains an important chemotherapeutic for treatment of different types of curable human malignancies, including germinative tumors, as well as Hodgkin’s lymphoma that commonly affect young individuals (4). However, a major limitation of BLM therapy in human is the development of pulmonary fibrosis and BLM pulmonary toxicity in up to 10–18% of patients receiving the drug (4–6). However, the pathogenesis of BLM-induced fibrosis and individual variation in susceptibility is not clearly understood.

In C57BL/6 (B6) mice, it has been reported that systemic or local administration of BLM induces an initial inflammatory reaction at day 7 (inflammatory phase), followed by fibrosis at day 21 (fibrotic phase) (7, 8). Currently, the pathogenesis of several human diseases could be clarified through leveraging of different animal models. For example, animal models of BLM-induced pulmonary fibrosis are useful for studying the mechanisms behind lung fibrosis as well as individual variation in susceptibility for lung fibrosis. Recently, BLM-induced lung injured mice was used as a model for human idiopathic pulmonary fibrosis to demonstrate different cellular senescence that mediates fibrotic pulmonary disease (9). Additionally, our previous report has shown significant positive correlations between immune cell infiltration into the lungs in two animal models of autoimmunity that develop a phenotype similar to human systemic lupus erythematosus (SLE) and the size of novel lymphoid clusters (LCs) associated with mediastinal fat tissues (MFTs) that termed as mediastinal fat-associated lymphoid cluster (MFALCs). Therefore, our previous studies suggested a possible role for such MFALCs in the progression of lung diseases (10, 11). However, further investigation is required to clarify the pathological role of MFALCs in lung diseases, especially those with an inflammatory nature.

The fat-associated LCs (FALCs) were firstly reported to be associated with the mesentery and the MFTs in human and healthy mice and were reported to have various roles in innate immunity (12, 13). Moreover, we reported strain-specific differences in the morphology of MFALCs among healthy strains of mice: Th1-biased B6, Th2-biased DBA/2Cr, and MRL/MpJ mice. Among these strains, the B6 mice presented significantly larger MFALCs (13). Interestingly, B6 mice were found to be susceptible to hypersensitive pneumonitis, a granulomatous inflammatory lung disease that affects both humans and mice. On the other hand, DBA/2 mice were resistant to this disease (14–16). Additionally, strain-specific differences in sensitivity to BLM-induced pulmonary fibrosis were revealed, where B6 mice were found to be more sensitive than Balb/c mice to pulmonary fibrosis (13). However, the mechanisms behind strain-specific differences in susceptibility to pulmonary fibrosis remain to be understood.

Recently, there are several approaches for attenuation or prevention of the BLM-induced lung injury and fibrosis in mice (17, 18). However, the pathogenesis of lung lesion progression and its correlation with the morphology of MFALCs remain unclear. Interestingly, it has been reported that inflammation-induced formation of mesenteric FALCs (19). Additionally, it has been revealed that the size and number of MFALCs increased profoundly at day 11 of filarial infection in mice (20). However, there are no reports regarding the morphology of MFALCs and their possible correlation with lung disease progression following inflammation in BLM-induced pneumonitis mouse models. Furthermore, leukocytes with ring shaped nuclei (ring cells) have been reported to comprise approximately 50% of fresh murine bone marrow cells; these cells have been identified in the peripheral blood as well as in different tissues during inflammation (21–23). However, their presence in the lungs and MFALCs during BLM-induced lung inflammations has not been examined.

In this report, we investigated the morphology of MFALCs following BLM-induced inflammation and hypothesized that they may play a major role in lung injury progression. We found that the size of MFALCs was significantly increased following BLM administration at both 7 days (inflammatory phase) and 21 days (fibrotic phase) as compared with phosphate-buffered saline (PBS)-treated control group. Moreover, significant higher ratios of both lymph vessels (LVs) and high endothelial venules (HEVs) in both MFALCs and lungs of the BLM group were found, when compared to those in the control group. The abundance of such vessels was positively correlated with MFALCs size. We conclude that MFALCs may have important roles in lung injury. In addition, it is possible that LVs and HEVs may provide potential avenues for cellular migration into and out of MFALCs, as well as into the lungs, thereby enhancing lung injuries and inducing disease progression.

## Materials and Methods

### Ethics Statement

The animal experiments were conducted with the approval of the Institutional Animal Care and Use Committee of the Graduate School of Veterinary Medicine, Hokkaido University (approval number; 15-0079). The investigators adhered to the Guide for the Care and Use of Laboratory Animals, Hokkaido University, Graduate School of Veterinary Medicine (approved by the Association for Assessment and Accreditation of Laboratory Animal Care International).

### Experimental Animals

Adult male B6 mice (12 weeks of age, 20–25 g) were used in the present study. Mice were purchased from Japan SLC, Inc. (Shizuoka, Japan). Animals were housed in standard laboratory cages with access to water and rodent laboratory chow *ad libitum*. The mice were maintained under specific pathogen-free conditions.

### Experimental Design

Bleomycin sulfate (LKT Laboratories, Inc., Lot Number: 2599253, catalog Number: B4518) was diluted with sterile PBS, and was administered at a single dose of 5 mg/kg in the BLM group. Control mice received sterile PBS. Each group consisted of 10 mice. Following anesthesia (0.3 mg/kg medetomidine, 4.0 mg/kg midazolam and 5.0 mg/kg butorphanol) administration, all groups received 50 µL intranasal (i.n.) instillations of either BLM or PBS on 0 days. On 7 days (early time point) and 21 days (late time point) after BLM or PBS instillation, five mice from each group were sacrificed; MFTs and lung samples were immediately collected and fixed in 4% paraformaldehyde. To enhance preservation of lung architecture, the lungs were first inflated with the fixative prior to immersion in the fixative solution. Two hours before sampling, mice were i.p. injected with 5-bromodeoxycytidine (BrdU) (Wako, Osaka, Japan) diluted in 0.01 M PBS, at a dose of 50 mg/kg.

### Tissue Preparation and Histopathological Analysis

**Figure 1:**
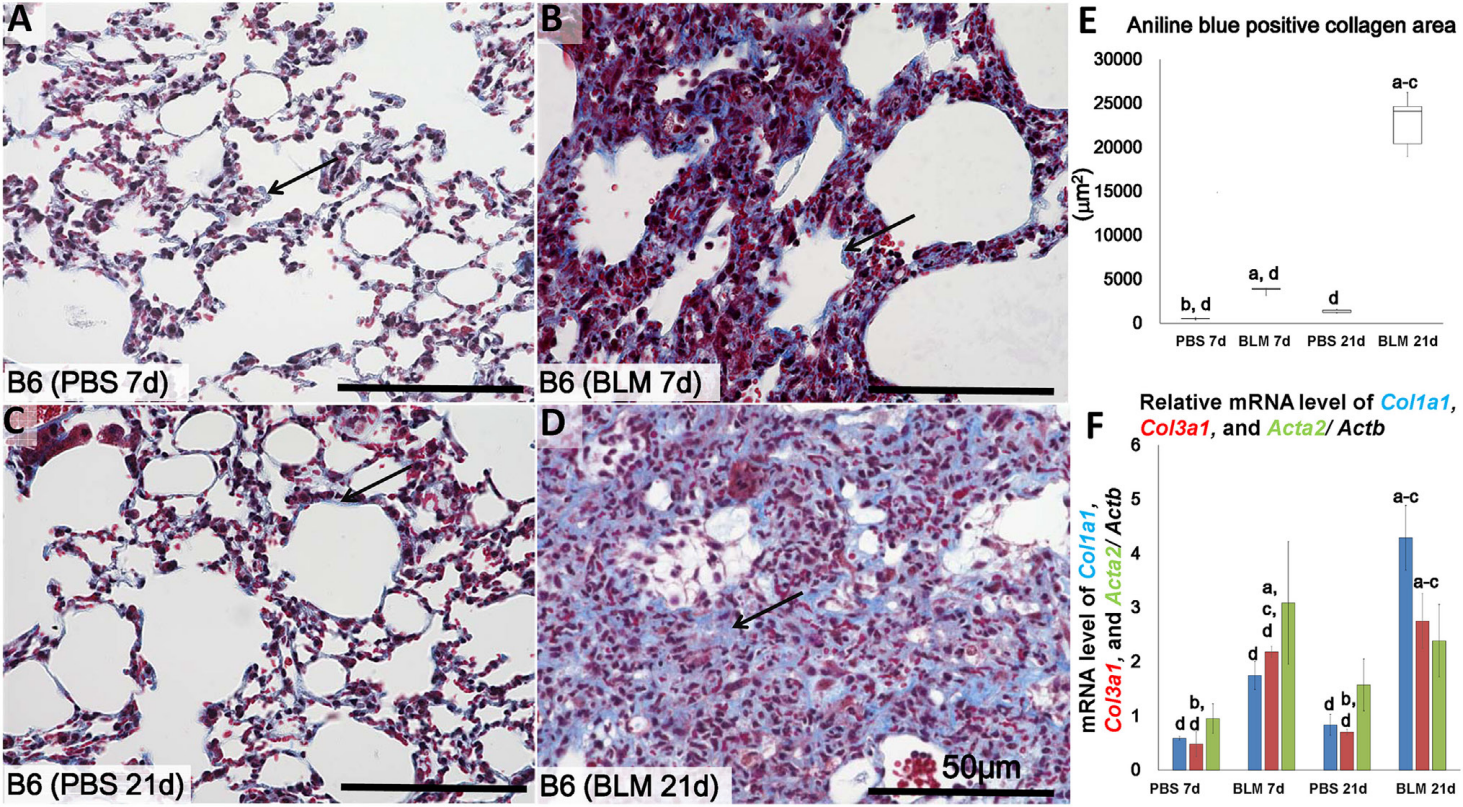
Identification of lung fibrosis in bleomycin (BLM) and phosphate-buffered saline (PBS) groups. Representative histopathological images of lung tissues stained with Masson’s trichrome at early stages (7 days after i.n. instillation of 50 µL of either PBS or 5 mg/kg BLM) **(A,B)**, respectively, and late stages (21 days after i.n. instillation of PBS or BLM) **(C,D)**, respectively. Arrows indicate collagen fibers. **(E)** Comparison of aniline blue^+^ collagen areas in lung tissues of different groups. **(F)** Quantitative expression of mRNA for collagen 1a1 (col1a1), collagen 3a1 (col3a1), and alpha-actin-2 (acta2)/*Actb*. The letters a, b, c, and d: significant differences between PBS group at 7 days (a), BLM group at 7 days (b), PBS group at 21 days (c), and BLM group at 21 days (d), analyzed by the Kruskal–Wallis test, followed by the Scheffé’s method (*P* < 0.05); *n* = 5 in each experimental group. Values are given as the mean ± SE.

Following overnight fixation at 4°C, specimens were washed, dehydrated in graded alcohol, and embedded in paraffin. Paraffin sections (3 µm) of MFT and lung tissues were prepared, stained with hematoxylin and eosin, and Masson’s trichrome (MT) (Figure 1). Samples were observed under a light microscope for histopathological analysis. Immunohistochemistry for CD3, B220, Iba1, Gr1, BrdU, peripheral node addressin (PNAd), and lymphatic vessel endothelial hyaluronic acid receptor 1 (LYVE-1) was performed to detect T-cells, B-cells, macrophages, granulocytes, proliferating cells, HEVs, and LVs, respectively. BrdU-incorporating cells were detected according to our previous report (13). Details of the antigen retrieval methods, staining conditions, and primary antibodies used are listed in Table 1. Briefly, following deparaffinization and antigen retrieval, sections were incubated with primary and secondary antibodies according to previously published streptavidin–biotin methods (24). Negative controls were incubated in 0.01 M PBS instead of primary antibody (data not shown).

**Table 1:** List of antibodies, working dilutions, and methods for antigen retrieval.

Antibody	Source	Dilution	Antigen retrieval	Heating condition
Rabbit anti-CD3	Nichirei (Tokyo, Japan)	1:200	Target retrieval solution (high pH) (produced by Dako, Japan)	105°C, 20 min
Rat anti-B220	Cedarlane (ON, Canada)	1:1,600	10 mM citrate buffer (pH 6.0)	105°C, 20 min
Rabbit anti-Iba1	Wako (Osaka, Japan)	1:1,200	10 mM citrate buffer (pH 6.0)	105°C, 20 min
Rat anti-Gr1	R and D system (MN, USA)	1:800	0.1% pepsin/0.2 N HCl	37°C, 5 min
Rat anti-BrdU	Abcam (Tokyo, Japan)	1:200	10 mM citrate buffer (pH 6.0)	105°C, 20 min
Rat anti-peripheral node addressin	BioLegend (San Diego, CA, USA)	1:500	Target retrieval solution (high pH) (produced by Dako, Japan)	105°C, 20 min
Rabbit anti-LYVE-1	Adipogen (San Diego, CA, USA)	1:500	10 mM citrate buffer (pH 6.0)	105°C, 20 min

### Real-time PCR Analysis

The total RNA was extracted from different frozen lung sample of each group by using Trizol (Thermo Fisher Scientific; Waltham, MA, USA). The total RNA of each sample was used as a template to synthesize cDNA using ReverTra Ace qPCR RT Master Mix (Toyobo; Osaka, Japan). The cDNA from each sample was analysed by TaqMan PCR method using probes for collagen, type I, alpha 1 (*Col1a1*), collagen, type III, alpha 1 (*Col3a1*), and actin, alpha 2, smooth muscle, aorta (*Acta2*), TaqMan Universal Master Mix II (Thermo Fisher Scientific), and PCR Machine (CFX Connect™ Real-Time PCR Detection System, Bio-Rad, CA, USA). The expression data were normalized to the expression levels of *Actb*.

### Isolation of Cells from MFT for Flow Cytometry

Mediastinal fat tissues were collected from BLM and PBS groups after transcardial perfusion with PBS. MFTs were chopped and digested in DMEM media containing 1 mg/ml Collagenase D (Roche, Tokyo, Japan) at 37°C for 40 min. The recovered solution was then filtered through nylon meshes. Cells were resuspended in 40% percoll and gently overlaid on 80% percoll. After centrifugation at 2,700 rpm for 15 min, viable cells were collected into DMEM media.

### Flow Cytometry and Cell Sorting

Single-cell suspensions were stained with fluorochrome-labeled monoclonal antibodies after incubation with FcR-blocking reagent (Miltenyi Biotec, Bergisch Gladbach, Germany). The following antibodies (all purchased from BioLegend) were used for flow cytometry: FITC-labeled anti-mouse CD11b, PE-labeled anti-mouse F4/80, PE/Cy7-labeled anti-mouse CD206, APC-labeled anti-mouse CD80, APC/Cy7-labeled anti-mouse CD45, FITC-labeled anti-mouse B220, PE-labeled anti-mouse CD3, PE/Cy7-labeled anti-mouse CD4, APC-labeled anti-mouse CD8, PE/Cy7-labeled anti-mouse CD138, APC-labeled anti-mouse CD19. Flow cytometric data were acquired on FACSVerse (BD Biosciences, San Jose, CA, USA) and then were analyzed using FlowJo software (TreeStar).

### Histoplanimetry

For histoplanimetry analysis, five mice were analyzed per group at both early and late time points. The spleen/body weight (BW) ratios were compared between the BLM and the PBS group at both 7 and 21 days. Digital images from H&E-, MT-, and immune-stained tissue sections were obtained using the BZ-X710 (Keyence, Osaka, Japan). Based on H&E-stained digital images, we calculated the ratio of LC area/total MFT area using the ImageJ software (ver. 1.32j, http://rsb.info.nih.gov/ij), as previously reported (13). The aniline blue^+^ fibrosis area (%) was measured from images of MT-stained section using the BZ-X710 fluorescent microscope (Keyence). From immune-stained digital images, positive cell counts for proteins of interest were determined *via* the BZ-X analyzer (Keyence). The percentages of B220^+^, CD3^+^, Gr1^+^, Iba1^+^, or BrdU^+^ cells in MFALCs were measured and compared among all groups using previously published methods (13). To determine the relative ratios of LYVE-1 + LVs to MFALC and lung area, we first measured total areas of LYVE-1 + LVs as well as the total field areas of MFALCs and the lungs using the ImageJ software. To calculate the relative ratio of the LYVE-1 + LVs areas, we divide the total areas of LYVE-1 + LVs by the total field areas; the average relative ratios in different fields were reported. The relative ratios of PNAd + HEVs in MFALCs were similar to that of LYVE-1 + Lvs. Additionally, the number of PNAd + HEVs in the lungs was manually counted in different lung lobes, and the average of the numbers within all lobes was reported.

### Statistical Analysis

All numerical results were presented as mean ± SE. Differences between groups were compared using the Kruskal–Wallis test. For multiple comparisons, the Scheffé’s method was applied, and statistical significance was set at *P* < 0.05. For comparison between two groups, Mann–Whitney *U-*test was performed (*P* < 0.05). Pearson’s correlation was used to determine the correlation between two variables (* significant value, *P* < 0.05, ** highly significant value, *P* < 0.01).

## Results

### Histopathological Features of the Lungs at Different Time Points in the BLM Mouse Model

Similar to methods employed in previous studies (6, 7), we examined the lungs at two points, 7 days (early time point) and 21 days (late time point) following i.n. BLM or PBS instillation in order to determine progression of lung injury and fibrosis. Analysis of MT-stained lung sections showed normal lung architecture in the control group at both 7 and 21 days (Figures 1A,C). However, the lungs in BLM-administered group showed large accumulations of mononuclear cells and reduced collagen fibers at 7 days (Figure 1B) post-instillation, and severe lung fibrosis at 21 days (Figure 1D). Significantly higher ratio of aniline blue-positive area was observed in the BLM groups as compared to the control group (Figure 1E). Furthermore, to analyze the degree of lung fibrosis, real-time PCR analysis was performed. The relative average of mRNA expression levels of *Col1a1, Col3a1*, and *Acta2*/*Actb* for each group was compared and statistically analyzed among other groups. *Col1a1* and *Col3a1* mRNA expressions were significantly higher in BLM group at 21 days than other groups. However, no significant difference was observed in that of *Acta2* among different groups (Figure 1F).

### Morphological Features of MFALCs in the BLM Mouse Model

**Figure 2:**
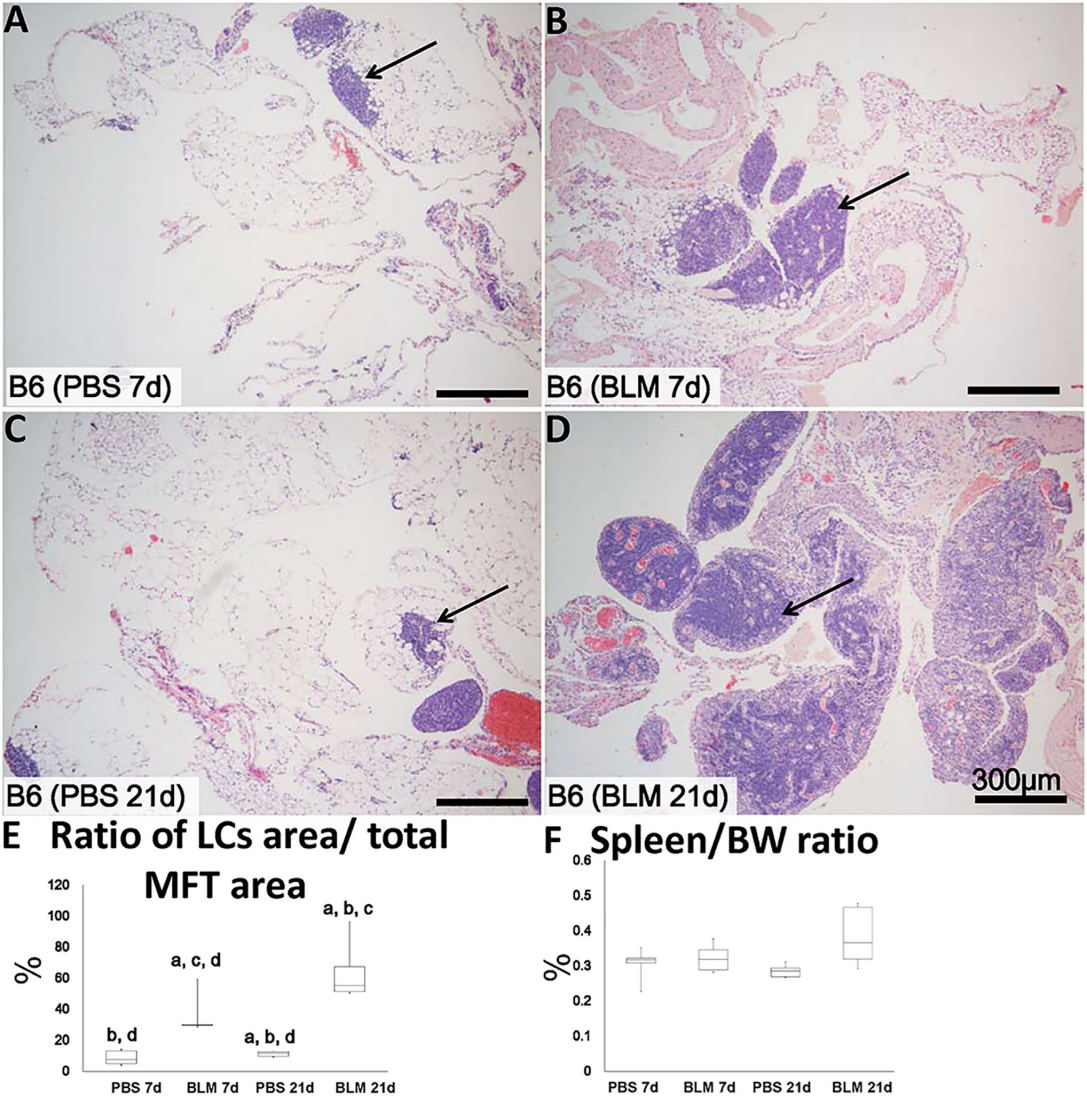
Mediastinal fat-associated lymphoid cluster (MFALC) development in Bleomycin (BLM)-induced pneumonitis model mice. H&E-stained mediastinal fat tissue (MFT) at 7 and 21 days following i.n. instillation of phosphate-buffered saline (PBS) **(A,C)** or 5 mg/kg BLM **(B,D)**. Arrows indicate MFALCs. **(E)** Ratio of LC area/total MFT area expressed as percentage. **(F)** Ratio of spleen/body weight expressed as percentage. The letters a, b, c, and d: significant differences between PBS group at 7 days (a), BLM group at 7 days (b), PBS group at 21 days (c), and BLM group at 21 days (d), analyzed by the Kruskal–Wallis test, followed by the Scheffé’s method (*P* < 0.05); *n* = 5 in each experimental group. Values = mean ± SE.

Analysis of H&E-stained sections indicated that the sizes of MFALCs were larger in the BLM group at both 7 and 21 days (Figures 2B,D, respectively) as compared to those in the PBS-treated control group (Figures 2A,C, respectively). To assess the histological index of MFALC development, we measured the LC/MFT area ratio. A significantly higher LC/MFT area ratio was observed in the BLM groups as compared to the control groups (Figure 2E). The spleen/BW ratios did not reveal significant differences between the two groups (Figure 2F).

### MFALC Immune Cell Populations and Lung Infiltration in the BLM Mouse Model

**Figure 3:**
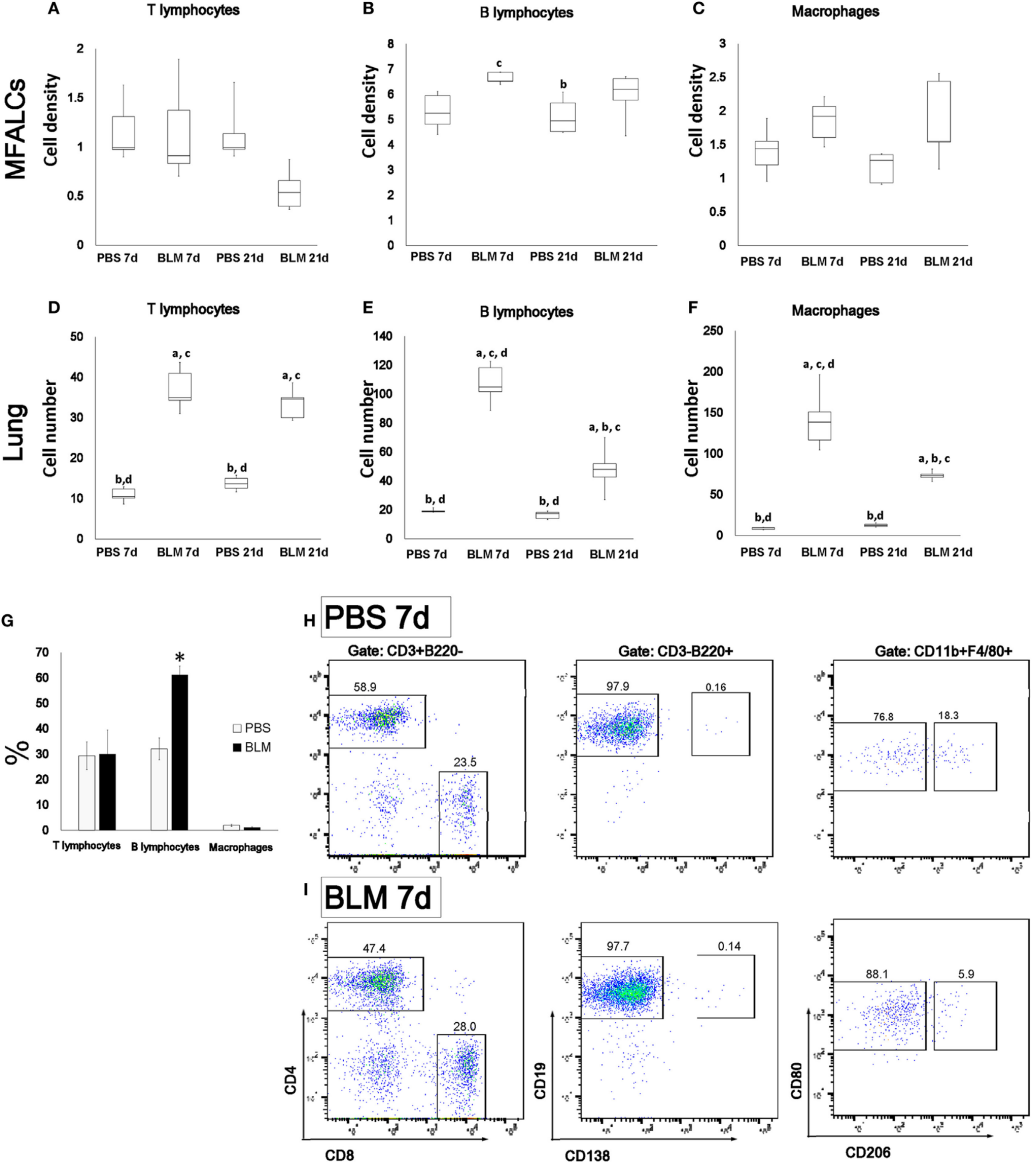
Immune cell populations in mediastinal fat-associated lymphoid clusters (MFALCs) and lungs of bleomycin (BLM)-induced pneumonitis. Densities of T-cells, B-cells, and macrophages in MFALCs **(A–C)**, and their average numbers in lung tissues **(D–F)**. The letters a, b, c, and d: significant differences between phosphate-buffered saline (PBS) group at 7 days (a), BLM group at 7 days (b), PBS group at 21 days (c), and BLM group at 21 days (d), analyzed by Kruskal–Wallis followed by Scheffé’s method (*P* < 0.05); *n* = 5 in each experimental group. Values = mean ± SE. The % of T-cells, B-cells, and macrophages in MFALCs were detected by flow cytometry among PBS and BLM groups at 7 days **(G)** (*n* ≥ 3 mice of each group). Statistically significant difference, as determined by the Mann–Whitney *U*-test (p < 0.05); comparison between the BLM and PBS groups at 7 days, with significance indicated by an asterisk (*). Values are shown as the mean ± SE. Flow cytometric analysis of T-cells (CD4/CD8), B-cells (CD19/CD138), and macrophages (M1/M2) subpopulations in MFALCs of PBS group **(H)**, and BLM group **(I)** at 7 days.

To characterize the cellular composition of MFALCs and lung infiltrates, immunohistochemical analysis for CD3, B220, Iba1, and Gr1 was performed to examine different subset of immune cells including T-cells, B-cells, macrophages, and granulocytes, respectively. The percentages of immune-positive cells in MFALCs were calculated and represented as cell densities. We also counted the number of immune-positive cells per lung field. In MFALCs, no significant difference was detected in T-cell density (Figure 3A), and macrophages (Figure 3C) between BLM and control groups. However, a significant difference in B-cell density was found between the BLM and control groups at 7 and 21 days (Figure 3B). Cellular infiltration of T- and B-cells, as well as macrophage into the lungs was significantly greater in the BLM group as compared to the control group at 7 and 21 days (Figures 3D–F). Moreover, the number of B-cell and macrophage infiltrates in the lungs of the BLM group at 7 days was significantly higher than that at 21 days. In accordance with immunohistochemical staining, flow cytometric analysis of the immune cells within MFALCs revealed a significant increase of B220^+^ B-cells in BLM group at day 7 when compared to PBS group (Figure 3G). In B-cells and T-cells subpopulations, there was no significant difference between the BLM and PBS groups, including CD4^+^ helper T-cells, CD8^+^ cytotoxic T-cells, CD19^+^ mature B-cells, and CD138^+^ plasma cells (Figures 3H,I). The macrophage subpopulations of BLM group showed a slight increase in the frequency of CD80^+^ M1 macrophages (Figures 3H,I).

**Figure 4:**
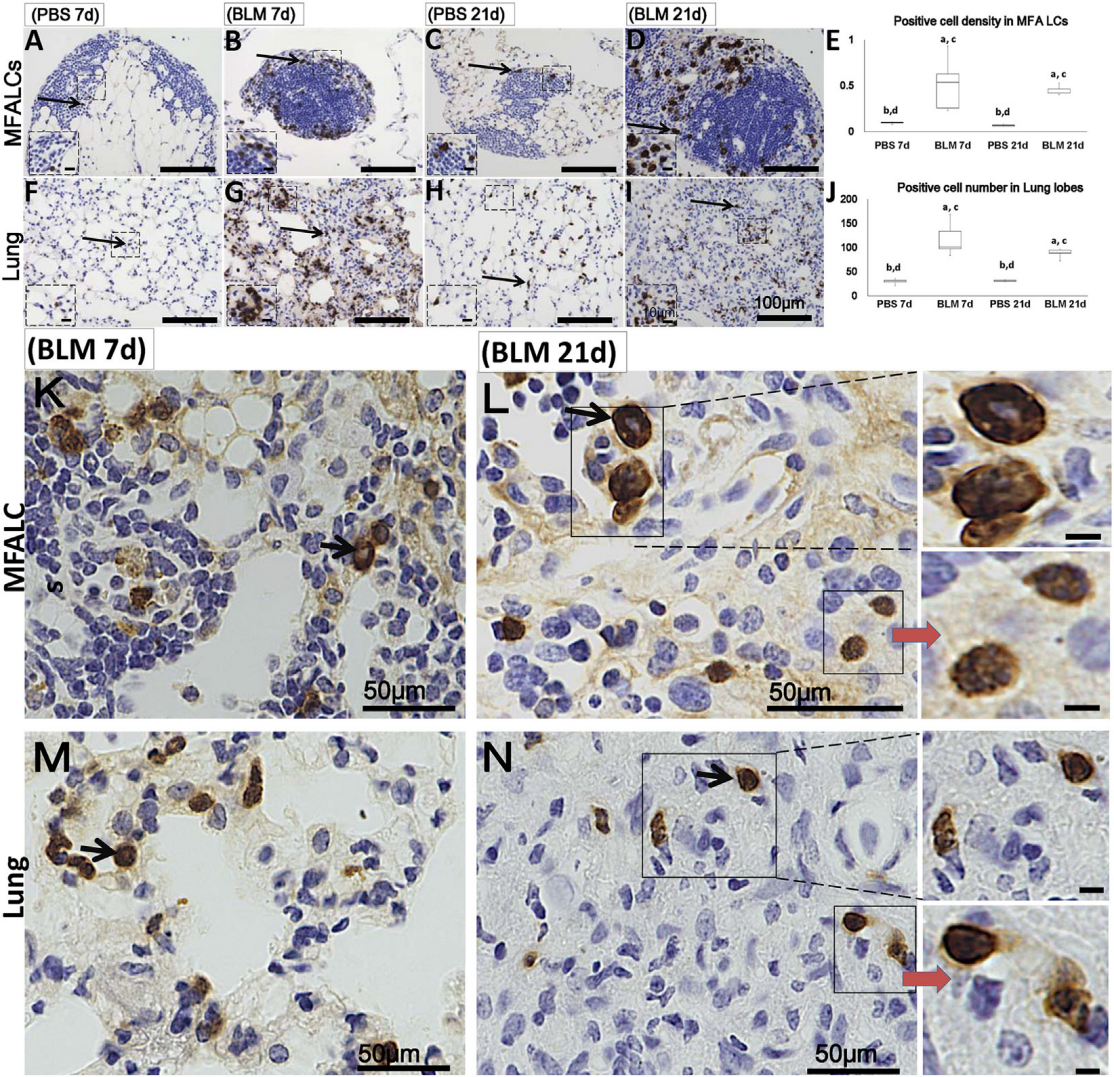
Granulocyte cell populations within mediastinal fat-associated lymphoid clusters (LCs) (MFALCs) and lung infiltrates in mouse model of bleomycin (BLM)-induced pneumonitis. Immunohistochemical images (low-power) show Gr-1^+^ cells in MFALCs **(A–D)** and lung tissues **(F–I)** of PBS group at 7 days, BLM group at 7 days, PBS group at 21 days, and BLM group at 21 days, respectively. **(E)** Gr-1^+^ cell density in MFALCs. **(J)** Average number of Gr-1^+^ cells in lung tissues. The letters a, b, c, and d: significant differences between PBS group at 7 days (a), BLM group at 7 days (b), PBS group at 21 days (c), and BLM group at 21 days (d), analyzed by the Kruskal–Wallis test, followed by the Scheffé’s method (*P* < 0.05); *n* = 5 in each experimental group. Values = mean ± SE. Immunohistochemical images (high power) show Gr-1^+^ cell in MFALCs **(K,L)** and lung tissues **(M,N)** of BLM groups at 7 and 21 days post-instillation. Some cells showed lobulated nucleus with complete or incomplete rings, others showed smooth non-lobulated ring shaped nucleus with central basophilic cytoplasm (arrows).

Additionally, numerous populations of Gr1^+^ cells were detected in both the MFALCs and the lungs of the BLM group. However, few Gr1^+^ populations were observed in the control group (Figures 4A–D,F–I). Gr1^+^ cell densities in the MFALCs and the lungs in the BLM group were revealed to be significantly higher as compared to those in the control group (Figures 4E,J). Interestingly, in the BLM group, Gr1^+^ cells showed varied nuclear surface and morphology. One population showed lobulated nucleus that were either complete or incomplete ring-shaped. Another population was larger in diameter and displayed a ring-shaped nucleus with smooth surface and central small basophilic cytoplasm (Figures 4K–N). The former population was rarely observed in either the MFALCs or the lungs of control mice.

### Proliferating Cells in the MFALCs and Lungs of BLM-Treated Mice

**Figure 5:**
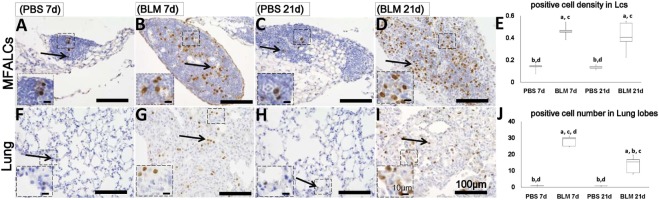
Proliferating cell populations in mediastinal fat-associated lymphoid clusters (LCs) (MFALCs) and lungs of Bleomycin (BLM)-induced pneumonitis mouse model. Immunohistochemical images show BrdU^+^ cell in MFALCs **(A–D)** and lung tissues **(F–I)** of phosphate-buffered saline (PBS) group at 7 days, BLM group at 7 days, PBS group at 21 days, and BLM group at 21 days, respectively. **(E)** Density of BrdU^+^ cells in MFALCs. **(J)** Average number of BrdU^+^ cells in lung tissues of different groups. The letters a, b, c, and d: significant differences between PBS group at 7 days (a), BLM group at 7 days (b), PBS group at 21 days (c), and BLM group at 21 days (d), analyzed by the Kruskal–Wallis test, followed by the Scheffé’s method (*P* < 0.05); *n* = 5 in each experimental group. Values = mean ± SE.

Numerous populations of BrdU^+^ cells were observed within the MFALCs and lungs of the BLM group (Figures 5B,D,G,I), but such populations were very few in the control group (Figures 5A,C,F,H). Statistical analysis showed that BrdU + cell densities in the MFALCs and the lungs were significant higher in the BLM group as compared to those in the control group (Figures 5E,J).

### Presence of LVs and HEVs in the MFALCs and Lungs of BLM-Treated Mice

**Figure 6:**
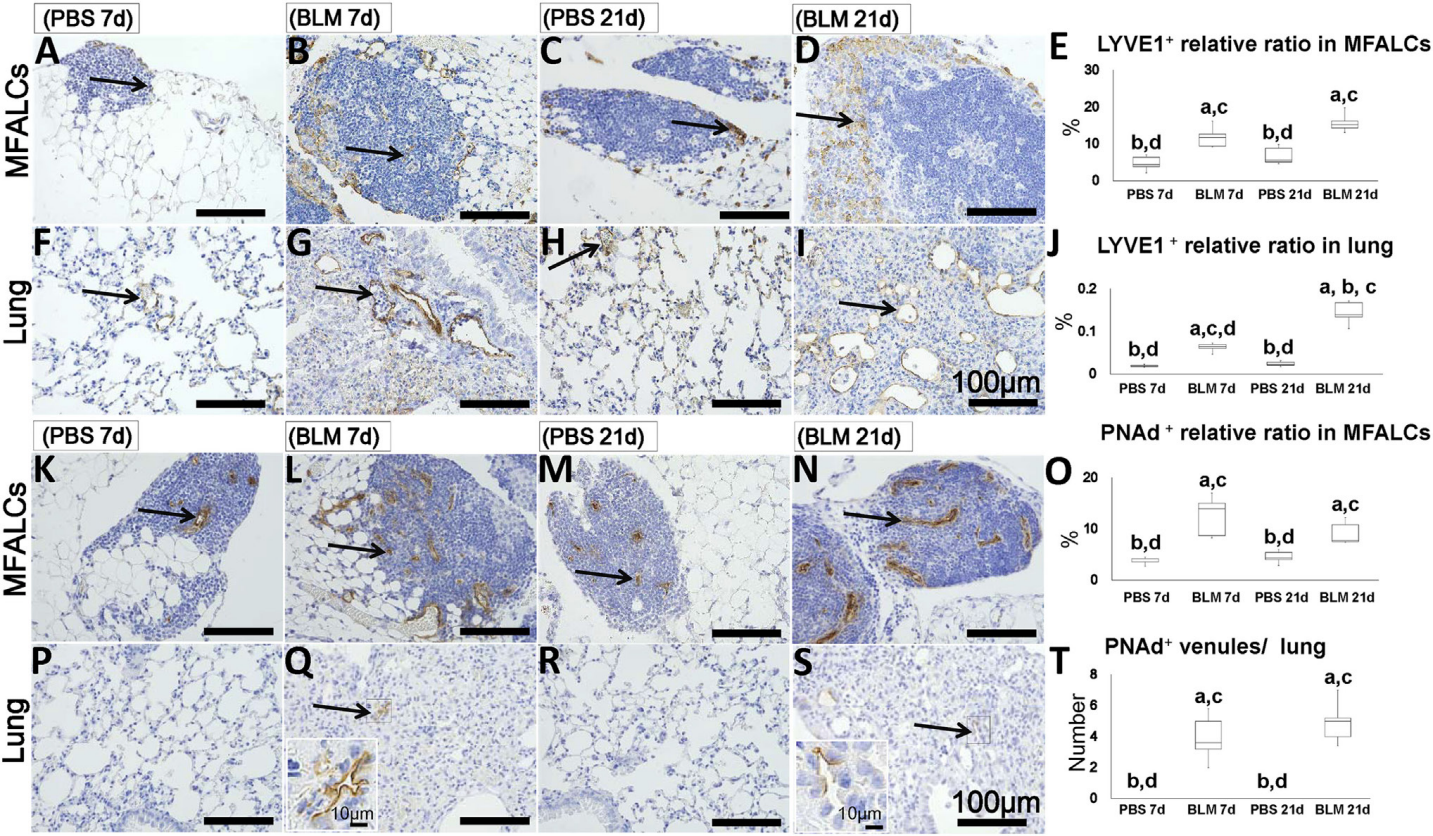
Presence of LVs and high endothelial venules (HEVs) in mediastinal fat-associated lymphoid clusters (LCs) (MFALCs) and lungs of Bleomycin (BLM)-induced pneumonitis in mice. Immunohistochemical images depict LYVE1^+^ LVs in MFALCs **(A–D)** and lung tissues **(F–I)** in phosphate-buffered saline (PBS) group at 7 days, BLM group at 7 days, PBS group at 21 days, and BLM group at 21 days, respectively. Arrows indicate LYVE1^+^ LVs in MFALCs **(A–D)** and lung tissues **(F–I)**. Percentage of LYVE1^+^ LVs in MFALCs **(E)** and lung tissues **(J)** of different groups. Immunohistochemical images of PNAd^+^ HEVs in the MFALCs **(K–N)** and lung tissue **(P–S)** are shown for PBS group at 7 days, BLM group at 7 days, PBS group at 21 days, and BLM group at 21 days, respectively. Arrows indicate PNAd^+^ HEVs in MFALCs **(K–N)** and lung tissues **(P–S)**. **(O)** Relative ratios of PNAd^+^ HEVs in the MFALCs. **(T)** Average number of PNAd^+^ HEVs in lung tissues. The letters a, b, c, and d: significant differences between PBS group at 7 days (a), BLM group at 7 days (b), PBS group at 21 days (c), and BLM group at 21 days (d), analyzed by the Kruskal–Wallis test, followed by the Scheffé’s method (*P* < 0.05); *n* = 5 in each experimental group. Values = mean ± SE.

To determine the abundance of LVs and HEVs in the MFALCs and the lungs, immunohistochemical analysis for LYVE-1 and PNAd was performed, respectively. LYVE-1^+^ LVs were more abundant in both the MFALCs and the lungs of BLM-administered mice (Figures 6B,D,G,I) as compared to those in the controls (Figures 6A,C,H,F). Quantitative measurements of the percentages of LVs in the MFALCs and the lungs revealed that BLM-treated group demonstrated significantly higher number of LVs as compared to the PBS-treated group at both 7 and 21 days post i.n. instillation (Figures 6E,J).

Similarly, the MFALCs in the BLM group yielded more developed PNAd + HEVs (Figures 6L,N) as compared to the PBS group (Figures 6K,M) at both 7 and 21 days. While we were able to detect PNAd^+^ HEVs in the lungs of the BLM group at both 7 and 21 days (Figures 6Q,S), we were unable to detect any PNAd^+^ HEVs in the lungs of the PBS group (Figures 6P,R). In additions, percentage of HEVs in MFALCs was greater in the BLM group as compared to the PBS group at both 7 and 21 days post i.n. instillation (Figure 6O). Finally, the lungs of the BLM group, but not the PBS group, showed elevated number of PNAd^+^ HEVs-like vessels at both 7 and 21 days post i.n. instillation (Figure 6T).

### Histopathological Correlations between MFALCs and BLM-Induced Lung Lesions in Mice

As shown in Table 2, we examined the pathological correlations between densities of CD3^+^, B220^+^, Iba1^+^, Gr1^+^, or BrdU^+^ proliferating cells in MFALCs and the number of cell infiltrates in the lungs in both treatment groups at 7 and 21 days. Except for CD3^+^ cells, cell densities of all investigated cell types in MFALCs were positively correlated with their infiltration into the lungs. However, the density of CD3^+^ cells in MFALCs was negatively correlated with that in the lungs. We also examined correlations between the quantitative indices of LV and HEVs in the MFALCs and those in the lungs at 7 and 21 days (Table 3). Results indicated that all investigated parameters in MFALCs were positively correlated with their corresponding parameters in the lungs. Moreover, we examined parameters that affected the development of MFALCs in both BLM and PBS groups at 7 and 21 days (Table 4). Correlation analysis was performed between ratios of MFALC/LV area or MFALC/HEV area and MFALC size. Results indicated that quantitative indices of LV and HEV were positively correlated with MFALC size.

**Table 2:** Correlations between density of immune-positive cells in the mediastinal fat-associated lymphoid clusters (MFALCs) and their numbers in the lung.

		BrdU	CD3	B220	Iba1	Gr1
MFALCs vs. lung	*r*	0.828**	−0.289	0.684**	0.459*	0.579**
	*P*	<0.001	0.217	0.001	0.042	0.007

*Significant, P < 0.05.

**Highly significant, P < 0.01.

*n = 20 for both phosphate-buffered saline and bleomycin groups*.

**Table 3:** Correlations between relative ratios of LVs and high endothelial venules (HEVs) in mediastinal fat-associated lymphoid clusters (MFALCs) and the lungs.

		LVs (LYVE1)	HEVs (peripheral node addressin)
MFALCs vs. lung	*r*	0.798**	0.642**
	*P*	<0.001	0.002

**Highly significant, P < 0.01.

*n = 20 for both phosphate-buffered saline and Bleomycin groups*.

**Table 4:** Correlation between area of mediastinal fat-associated lymphoid clusters (MFALCs) and the relative ratios of LVs or high endothelial venules (HEVs) in the MFALCs.

		LVs (LYVE1)	HEVs (peripheral node addressin)
MFALCs vs. vessels areas	*r*	0.734**	0.475*
	*P*	<0.001	0.034

*Significant, P < 0.05.

**Highly significant, P < 0.01.

*n = 20 for both phosphate-buffered saline and Bleomycin groups*.

## Discussion

Bleomycin is a chemotherapeutic antibiotic that is used as an effective antineoplastic drug. However, repeated systemic administration of BLM may result in lung inflammation and fibrosis (25). This side effect was reported to occur in 10% of patients receiving the BLM treatment (26–28). Increased knowledge of the pathogenesis of BLM-induced lung injury may lead to development of agents capable of preventing or treating established BLM-induced pneumonitis. Due to the difficulties in studying the pathogenesis of lung fibrosis in humans, different animal models of pulmonary fibrosis have been developed to investigate potential therapies for IPF (9). BLM-induced lung fibrosis has been studied in many experimental models including dogs (29), hamsters (30), rats (31), and in mice (7, 32) to reveal the cellular and molecular basis for lung fibrosis. The time course of BLM-induced fibrosis was revealed (33). Lung lesions have been shown to occur in two stages following intratracheal BLM instillation: inflammatory and fibrotic at 7 and 21 days, respectively.

Notably, in the murine model, remarkable strain-specific differences were observed in susceptibility to fibrosis following BLM administration, with CBA and B6 mice being highly susceptible, and Balb/c mice being relatively fibrosis-resistant. These differences are likely due to different expression patterns of cytokines and proteases/anti-proteases (8, 33). We have previously revealed strain-specific differences in the size of a novel LC associated with the MFTs; B6 mice showed more developed MFALCs than other mouse strains (13). In a recent study, we demonstrated better developed MFALCs in two mic model of autoimmune disease (MRL/MpJ-lpr and BXSB/MpJ-Yaa) that show a phenotype similar to human SLE as compared to control strains. In addition, we reported significantly positive correlation between the size of LCs and lung infiltration in mice with autoimmune diseases, suggesting that MFALCs may play a role in progression of lung lesions (10). Moreover, it has been recently revealed that the size of mesenteric FALCs increases following induction of peritonitis in mice (19). Also, it has been reported that the number and size of the CD45^+^ MFALCs following parasitic infection of the pleura and lung inflammation were significantly increased (20). However, to date, the pathogenesis of lung inflammation and fibrosis is still not clear. The association between BLM-induced lung inflammation and development of MFALCs is also another unexplored topic. Recent studies revealed that it is not possible to address the direct role of pleural FALCs in protection against nematode infection due to it is not practical to remove FALCs from the pleural space (20). Furthermore, through our morphological and histological observations of the MFTs, we could detect many connections extending from the MFTs into the lung and thymus (data not shown). Subsequently, we suggested that from the practical point of view, it is difficult to remove the MFALCs to address their direct role in lung diseases progression. Therefore, in the current study, we wanted to determine the relationship between MFALCs development and sensitivity to pneumonitis following BLM administration in comparison of the PBS control administered group.

In this study, we examined the histopathological changes in both the lungs and MFALCs of B6 mice at 7 and 21 days following i.n. instillation of BLM. Similar to other reports (8, 33), the lungs of mice in the BLM group showed inflammatory and fibrotic changes at 7 and 21 days, respectively. Interestingly, our results revealed that MFALCs was significantly larger in the BLM group at both 7 and 21 days, indicating that inflammatory and fibrotic reactions in the lungs were rapidly induced by MFALC activation. This was in agreement with a recent observation, where sizes of FALCs in the mediastinum and pericardium were shown to be profoundly increased 11 days following filarial infection (20). The present study also revealed significantly higher BrdU + proliferating cells in MFALCs of the BLM group as compared to those in the control group. These data suggest that the BLM induces proliferation of cells that form MFALCs.

We characterized the cellular compositions of MFALCs and lung infiltrates in both the BLM and control groups. Higher cell densities and greater numbers of immune cells, especially B-cells, macrophages, and granulocytes, were observed in both MFALCs and lungs of the BLM group as compared to those of the control group. Interestingly, our flow cytometric analysis revealed an obvious enrichment of B-cells in MFALCs after treatment with BLM. This was in agreement with previous observations, where B-cells were found to be significantly upregulated in pericardial FALCs upon infection by *Litomosoides sigmodontis*, a parasite that is restricted to the pleural cavity in its first stages of development (20, 34). We also showed significantly positive correlations between quantitative parameters of immune cells (B-cells, macrophages, and granulocytes) in both the lungs and the MFALCs. Therefore, MFALCs may be essential in the progression of lung diseases, especially those inflammatory in nature. On the other hand, we saw a decrease in T-cells density in MFALCs following BLM administration, which was negatively correlated with lung infiltration. It is possible that changes in the number of other cell types such as B-cells and macrophages 21 days post-BLM treatment affect T-cell density. Together, these results suggest that inflammation could induce proliferation of B-cells, macrophages, and granulocytes (with the exception of T-cells) within the MFALCs and lungs following BLM exposure. Previous studies have also demonstrated proliferation and differentiation of B-cells in mesenteric FALCs following induction of peritonitis in mice (19). Interestingly, it has been reported that following both lung infection with nematode larvae and lung inflammation by fungal allergen inhalation, the MFALCs stromal cells produce IL-33, which lead to activation of type 2 innate lymphoid cells and IL-5 secretion. This in turn stimulates the recruitment and activation of B cells (20).

Interestingly, our results revealed the presence of Gr1+ cells with heterogeneous nuclear surface and morphology. Some populations showed lobulated nucleus that form either complete or incomplete ring shapes. Another population exhibited larger diameters and contained a ring-shaped nucleus with smooth surface. A previous study termed leukocytes with ring-shaped nuclei ring cells (35). These cells comprised 50% of bone marrow cells and were also present in the peripheral blood and were part of inflammatory infiltrates in mice. Furthermore, these cells were also reported to be present in the peripheral blood of human patients with infectious mononucleosis (36) and myeloproliferative diseases (37–39). Non-segmented ring cells were named mononuclear-like ring cells. As shown by electron micrographs as well as histochemical and immunohistochemical staining, these cells exhibit the typical characteristics of mononuclear phagocytes, monocytes, promonocytes, and promyelocytes (35). Interestingly, monocyte-derived alveolar macrophages (Mo-AMs) were recently reported in the lung tissue in addition to the tissue-resident alveolar macrophages (TR-AMs) and their number increased following BLM-induced lung injury (40, 41). Furthermore, it has been revealed that the Mo-AMs play role in the progression of lung fibrosis, whereas genetic deletion of Mo-AMs following their recruitment to the lung ameliorated lung fibrosis. However, TR-AMs did not have role in fibrosis development (41).

It is known that the first step of lymphocyte migration into tissues is their adhesion to the vascular endothelium. In the lymphoid organs, lymphocyte adherence and transendothelial migration occur at HEVs, which act as sites for T-cells and B-cell entry (42). In humans, HEVs are found in all secondary lymphoid organs including lymph nodes, tonsils, and Peyer’s patches. Additionally, HEV-like vessels were also observed in chronically inflamed non-lymphoid tissues and were suggested to support the recruitment of lymphocyte into these sites (43). Furthermore, HEVs are also observed within the LCs of the omentum as a specialized type of post-capillary venules important for lymphocyte trafficking (44, 45). The abundance of HEVs in various tissue types suggests that they may contribute to the pathogenesis of many chronic inflammatory diseases in both human and animals (46). In non-obese diabetic mice, HEVs have been detected in the inflamed pancreas, as well as in the salivary glands (47). Areas of dense lymphocytic infiltration containing HEVs were observed in cases of chronic gut inflammation, such as in Crohn’s disease and ulcerative colitis. They were also associated with autoimmune thyroiditis such as Graves’ disease and Hashimoto’s thyroiditis (48–50).

In the present study, one of our aims was to investigate the possible role of LVs and HEVs in the lungs during immune cell infiltration. To this end, we examined the distribution of LVs and HEVs in MFALCs and lung tissues in both BLM and control groups. LYVE-1 + LVs were found to be significantly higher in both MFALCs and lung tissues of BLM-treated group as compared to those in the control group. Well-developed PNAd + HEVs were also observed to be more numerous in the MFALCs of BLM-treated group as compared to the control group. Interestingly, PNAd + HEVs were found in the lungs of BLM-treated group, but not in the control group, at both 7 and 21 days following drug administration. Furthermore, our results revealed higher percentage of B-cell populations following BLM administration suggesting their proliferation and or recruitment. Therefore, we suggest that B-cell response and HEVs may be essential to the development of inflammation and fibrosis following BLM administration. However, further investigations are required to expand our understanding of the factors associated with the development of HEVs in lung tissues post-BLM treatment. Manipulation of these factors could provide novel therapeutic treatments for many inflammatory lung diseases in both humans and animals.

## Author Contributions

YE conceived and designed the experiments, interpreted data, and wrote the manuscript. OI designed the experiments and edited the manuscript. KT provided biological resources and expert for FACs analysis experiment and analyzed the data. TN and MM conceived experiments and took care of mice. YK conceptualized the research, performed data interpretation, contributed to the discussion, and reviewed the manuscript.

## Conflict of Interest Statement

This manuscript has not been published or presented elsewhere in part or in entirety, and not under consideration by another journal. All the authors declare no competing financial interests.

## References

[1] Raghu G Amatto VC Behr J Stowasser S. Comorbidities in idiopathic pulmonary fibrosis patients: a systematic literature review. Eur Respir J (2015) 46:1113–30. 10.1183/13993003.02316-2014 26424523

[2] Hutchinson J Fogarty A Hubbard R McKeever T. Global incidence and mortality of idiopathic pulmonary fibrosis: a systematic review. Eur Respir J (2015) 46:795–806. 10.1183/09031936.00185114 25976683

[3] Williamson JD Sadofsky LR Hart SP. The pathogenesis of bleomycin-induced lung injury in animals and its applicability to human idiopathic pulmonary fibrosis. Exp Lung Res (2015) 41(2):57–73. 10.3109/01902148.2014.979516 25514507

[4] Reinert T Baldotto CSR Nunes FAP Scheliga AAdS Bleomycin-induced lung injury. J Cancer Res (2013) 2013:1–9. 10.1155/2013/480608

[5] Meadors M Floyd J Perry MC Pulmonary toxicity of chemotherapy. Semin Oncol (2006) 33:98–105. 10.1053/j.seminoncol.2005.11.005 16473648

[6] Martin WG Ristow KM Habermann TM Colgan JP Witzig TE Ansell SM Bleomycin pulmonary toxicity has a negative impact on the outcome of patients with Hodgkin’s lymphoma. J Clin Oncol (2005) 23:30 10.1200/JCO.2005.02.7243 16186594

[7] Izbicki G Segel MJ Christensen TG Conner MW Breuer R. Time course of bleomycin-induced lung fibrosis. Int J Exp Pathol (2002) 83:111–9. 10.1046/j.1365-2613.2002.00220.x 12383190 PMC2517673

[8] Manali ED Moschos C Triantafillidou C Kotanidou A Psallidas I Karabela SP Static and dynamic mechanics of the murine lung after intratracheal bleomycin. BMC Pulm Med (2011) 11:33 10.1186/1471-2466-11-33 21627835 PMC3128859

[9] Schafer MJ White TA Iijima K Haak AJ Ligresti G Atkinson EJ Cellular senescence mediates fibrotic pulmonary disease. Nat Commun (2017) 8:14532. 10.1038/ncomms14532 28230051 PMC5331226

[10] Elewa YH Ichii O Kon Y. Comparative analysis of mediastinal fat-associated lymphoid cluster development and lung cellular infiltration in murine autoimmune disease models and the corresponding normal control strains. Immunology (2016) 147:30–40. 10.1111/imm.12539 26439309 PMC4693879

[11] Elewa YH Ichii O Kon Y. Sex-related differences in autoimmune-induced lung lesions in MRL/MpJ-fas^lpr^ mice are mediated by the development of mediastinal fat-associated lymphoid clusters. Autoimmunity (2017) 50:306–16. 10.1080/08916934.2017.1344973 28665157

[12] Moro K Yamada T Tanabe M Takeuchi T Ikawa T Kawamoto H Innate production of T(H)2 cytokines by adipose tissue-associated c-Kit (+) Sca-1(+) lymphoid cells. Nature (2010) 463:540–4. 10.1038/nature08636 20023630

[13] Elewa YH Ichii O Otsuka S Hashimoto Y Kon Y. Characterization of mouse mediastinal fat-associated lymphoid clusters. Cell Tissue Res (2014) 357:731–41. 10.1007/s00441-014-1889-6 24853670

[14] Fink JN Hypersensitivity pneumonitis. Clin Chest Med (1992) 13:303–9. 1511555

[15] Sharma OP Fujimura N. Hypersensitivity pneumonitis: a noninfectious granulomatosis. Semin Respir Infect (1995) 10:96–106. 7569404

[16] Butler NS Monick MM Yarovinsky TO Powers LS Hunninghake GW. Altered IL-4 mRNA stability correlates with Th1 and Th2 bias and susceptibility to hypersensitivity pneumonitis in two inbred strains of mice. J Immunol (2002) 169:3700–9. 10.4049/jimmunol.169.7.3700 12244163

[17] Yokoyama T Yanagihara T Suzuki K Hamada N Tsubouchi K Ogata-Suetsugu S Depletion of club cells attenuates bleomycin-induced lung injury and fibrosis in mice. J Inflamm (Lond) (2017) 14:20. 10.1186/s12950-017-0168-1 28936122 PMC5604393

[18] Liu M-H Lin A-H Ko H-K Perng D-W Lee T-S Kou YR. Prevention of bleomycin-induced pulmonary inflammation and fibrosis in mice by paeonol. Front Physiol (2017) 8:193. 10.3389/fphys.2017.00193 28408888 PMC5374202

[19] Bénézech C Luu NT Walker JA Kruglov AA Loo Y Nakamura K Inflammation-induced formation of fat-associated lymphoid clusters. Nat Immunol (2015) 16:819–28. 10.1038/ni.3215 26147686 PMC4512620

[20] Jackson-Jones LH Duncan SM Magalhaes MS Campbell SM Maizels RM McSorley HJ Fat-associated lymphoid clusters control local IgM secretion during pleural infection and lung inflammation. Nat Commun (2016) 7:12651. 10.1038/ncomms12651 27582256 PMC5025788

[21] Sunderkötter C Beil W Roth J Sorg C. Cellular events associated with inflammatory angiogenesis in the mouse cornea. Am J Pathol (1991) 138:931–9. 1707239 PMC1886108

[22] Roth J Sunderkötter C Goebeler M Gutwald J Sorg C. Expression of the calcium-binding proteins MRP8 and MRP14 by early infiltrating cells in experimental contact dermatitis. Int Arch Allergy Immunol (1992) 98:140–5. 10.1159/000236177 1643439

[23] Sunderkötter C Kunz M Steinbrink K Meinardus-Hager G Goebeler M Bildau H Resistance of mice to experimental leishmaniasis is associated with more rapid appearance of mature macrophages in vitro and in vivo. J Immunol (1993) 151:4891–901. 8409447

[24] Elewa YH Bareedy MH Abuel-Atta AA Ichii O Otsuka S Kanazawa T Structural characteristics of goat (*Capra hircus*) parotid salivary glands. Jpn J Vet Res (2010) 58:121–35. 10.14943/jjvr.58.2.121 20715422

[25] Breuer R Lossos IS Or R Krymsky M Dagan A Yedgar S. Abatement of bleomycin-induced pulmonary injury by cell-impermeable inhibitor of phospholipase A2. Life Sci (1995) 57:L237–40. 10.1016/0024-3205(95)02116-Z 7564888

[26] Jules-Elysee K White DA. Bleomycin-induced pulmonary toxicity. Clin Chest Med (1990) 11:1–20. 1691067

[27] Sleijfer S Bleomycin-induced pneumonitis. Chest (2001) 120:617–24. 10.1378/chest.120.2.617 11502668

[28] O’Sullivan JM Huddart RA Norman AR Nicholls J Dearnaley DP Horwich A. Predicting the risk of bleomycin lung toxicity in patients with germ-cell tumours. Ann Oncol (2003) 14:91–6. 10.1093/annonc/mdg020 12488299

[29] Fleischman RW Baber JR Thompson GR Schaeppi UH Illievski VR Cooney DA Bleomycin induced interstitial pneumonia in dogs. Thorax (1971) 26:675–82. 10.1136/thx.26.6.675 4111464 PMC472381

[30] Snider GL Celli BR Goldstein RH O’Brien JJ Lucey EC Chronic interstitial pulmonary fibrosis produced in hamsters by endotracheal bleomycin. Lung volumes, volume-pressure relations, carbon monoxide uptake, and arterial blood gas studied. Am Rev Respir Dis (1978) 117:289–97. 10.1164/arrd.1978.117.2.289 76453

[31] Thrall RS McCormick JR Jack RM McReynolds RA Ward PA. Bleomycin-induced pulmonary fibrosis in the rat: inhibition by indomethacin. Am J Pathol (1979) 95:117–30. 86304 PMC2042298

[32] Liu W Wan J Han J-Z Li C Feng D-D Yue S-J Antiflammin-1 attenuates bleomycin-induced pulmonary fibrosis in mice. Respir Res (2013) 14:101. 10.1186/1465-9921-14-101 24098933 PMC3856527

[33] Phan SH Kunkel LK. Lung cytokine production in bleomycin-induced pulmonary fibrosis. Exp Lung Res (1992) 18:29–43. 10.3109/01902149209020649 1374023

[34] Hoffmanna W Petitc G Schulz-Keya H Taylorb D Bainc O Le Goff L. *Litomosoides sigmodontis* in mice: reappraisal of an old model for filarial research. Parasitol Today (2000) 16:387–9. 10.1016/S0169-4758(00)01738-5 10951598

[35] Biermann H Pietz B Dreier R Schmid KW Sorg C Sunderkötter C Murine leukocytes with ring-shaped nuclei include granulocytes, monocytes, and their precursors. J Leukoc Biol (1999) 65:217–31. 10.1002/jlb.65.2.217 10088605

[36] Peichev M Ring cells in infectious mononucleosis. Br J Haematol (1986) 62:397–8. 10.1111/j.1365-2141.1986.tb02945.x 3947554

[37] Stavem P Hjort PF Vogt E van der Hagen CB Ring-shaped nuclei of granulocytes in a patient with acute erythroleukaemia. Scand J Haematol (1969) 6:31–2. 10.1111/j.1600-0609.1969.tb01798.x 5257575

[38] Langenhuijsen MM. Neutrophils with ring-shaped nuclei in myeloproliferative disease. Br J Haematol (1984) 58:227–30. 10.1111/j.1365-2141.1984.tb06080.x 6591944

[39] Kanoh T Saigo K Yamagishi M. Neutrophils with ring-shaped nuclei in chronic neutrophilic leukemia. Am J Clin Pathol (1986) 86:748–51. 10.1093/ajcp/86.6.748 3466524

[40] Misharin AV Morales-Nebreda L Mutlu GM Budinger GR Perlman H Flow cytometric analysis of macrophages and dendritic cell subsets in the mouse lung. Am J Respir Cell Mol Biol (2013) 49:503–10. 10.1165/rcmb.2013-0086MA 23672262 PMC3824047

[41] Misharin AV Morales-Nebreda L Reyfman PA Cuda CM Walter JM McQuattie-Pimentel AC Monocyte-derived alveolar macrophages drive lung fibrosis and persist in the lung over the life span. J Exp Med (2017) 214(8):1–18. 10.1084/jem.20162152 28694385 PMC5551573

[42] Westermann J Blaschke V Zimmermann G Hirschfeld U Pabst R Random entry of circulating lymphocyte subsets into peripheral lymph nodes and Peyer’s patches: no evidence in vivo of a tissue-specific migration of B and T cells at the level of high endothelial venules. Eur J Immunol (1992) 22:2219–23. 10.1002/eji.1830220906 1516614

[43] Freemont AJ. Functional and biosynthetic changes in endothelial cells of vessels in chronically inflamed tissues: evidence for endothelial control of lymphocyte entry into diseased tissues. J Pathol (1988) 155:225–30. 10.1002/path.1711550308 3411383

[44] Rangel-Moreno J Moyron-Quiroz JE Carragher DM Kusser K Hartson L Moquin A Omental milky spots develop in the absence of lymphoid tissue-inducer cells and support B and T cell responses to peritoneal antigens. Immunity (2009) 30:731–43. 10.1016/j.immuni.2009.03.014 19427241 PMC2754314

[45] Buscher K Wang H Zhang X Striewski P Wirth B Saggu G Protection from septic peritonitis by rapid neutrophil recruitment through omental high endothelial venules. Nat Commun (2016) 7:10828 10.1038/ncomms10828 26940548 PMC4785224

[46] Girard JP Springer A. High endothelial venules (HEVs): specialized endothelium for lymphocyte migration. Immunol Today (1995) 16:449–57. 10.1016/0167-5699(95)80023-9 7546210

[47] Faveeuw C Gagnerault MC Lepault F. Expression of homing and adhesion molecules in infiltrated islets of Langerhans and salivary glands of nonobese diabetic mice. J Immunol (1994) 152:5969–78. 8207221

[48] Duijvestijn AM Horst E Pals ST Rouse BN Steer AC Picker LJ High endothelial differentiation in human lymphoid and inflammatory tissues defined by monoclonal antibody HECA-452. Am J Pathol (1994) 130:147–55. 3276207 PMC1880545

[49] Michie SA Streeter PR Bolt PA Butcher EC Picker LJ. The human peripheral lymph node vascular addressin. An inducible endothelial antigen involved in lymphocyte homing. Am J Pathol (1993) 143:1688–98. 8256856 PMC1887255

[50] Salmi M Granfors K MacDermott R Jalkanen S. Aberrant binding of lamina propria lymphocytes to vascular endothelium in inflammatory bowel diseases. Gastroenterology (1994) 106:595–605. 10.1016/0016-5085(94)90691-2 8119529

